# Incurable Insanity

**Published:** 1854-12

**Authors:** 


### INCURABLE INSANITY.

To be hopelessly insane, how terrible ; a worse calamity than death itself. There are some singular facts in reference to insanity. New England gives the largest per centage of all sections of the Union, and the extreme Southern States the least. In some Northern localities, there is one insane person to about every six hundred of the population, while in the South, and especially among the negroes, there is not one in six thousand.

Another striking fact is, that the asylums in this country have a larger number of inmates from among the farming population than from among any other calling. This is accounted for in the sameness, the horsemill life of a farmer; he trudges around in the same track from one decade to another, bringing into requisition a single set of mental energies, while all the rest remain dormant to a certain extent and grow wild like an undisturbed field. The fact is, no man was ever made to be a loafer, not even as to a part of his faculties, corporeal, mental or moral. There is enough to do in these ages of the world, to keep every son and daughter of Adam at work all the time of his waking existence, not at mind-work alone or body-work alone, but mind and body both at work all the time of working hours. It is because of the partial loaferism of the multitude that so many of the truly good among us perish before their time. Often is it that when men find a competent and willing worker they impose on him the labor of a dozen men, and the inevitable result is that in a few years he is literally worked to death. We have had a near and recent instance of this in the untimely death of Bishop Wainwright.

The true lesson is, let the multitude do more and the few less, then will not these few die before their time, and then too will not the multitude overcrowd our lunatic asylums as they now do, no less than five hundred and sixty-one being in a single hospital in Massachusetts.

